# Newborn screening for spinal muscular atrophy in Germany: clinical results after 2 years

**DOI:** 10.1186/s13023-021-01783-8

**Published:** 2021-03-31

**Authors:** Katharina Vill, Oliver Schwartz, Astrid Blaschek, Dieter Gläser, Uta Nennstiel, Brunhilde Wirth, Siegfried Burggraf, Wulf Röschinger, Marc Becker, Ludwig Czibere, Jürgen Durner, Katja Eggermann, Bernhard Olgemöller, Erik Harms, Ulrike Schara, Heike Kölbel, Wolfgang Müller-Felber

**Affiliations:** 1 Dr. v. Hauner Children’s Hospital, Department of Pediatric Neurology and Developmental Medicine, LMU – University of Munich, Lindwurmstraße 4, 80337 München, Germany; 2 Department of Pediatric Neurology, Münster University Hospital, Münster, Germany; 3 Center for Human Genetics, Genetikum®, Neu-Ulm, Germany; 4 Screening Center of the Bavarian Health and Food Safety Authority, Oberschleißheim, Germany; 5 Institute of Human Genetics, Center for Molecular Genetics Cologne and Center for Rare Diseases, University of Cologne, Cologne, Germany; 6 Labor Becker und Kollegen, Munich, Germany; 7 Department of Operative/Restorative Dentistry, Periodontology and Pedodontics, LMU – University of Munich, München, Germany; 8 Institute of Human Genetics, Medical Faculty, RWTH Aachen University, Aachen, Germany; 9 Formerly Labor Becker, Olgemöller und Kollegen, Munich, Germany; 10 Department of Pediatrics, Muenster University Hospital, Münster, Germany; 11 Department of Pediatric Neurology, Developmental Neurology and Social Pediatrics, University of Essen, Essen, Germany

## Abstract

**Background:**

Spinal muscular atrophy (SMA) is the most common neurodegenerative disease in childhood. Since motor neuron injury is usually not reversible, early diagnosis and treatment are essential to prevent major disability. Our objective was to assess the impact of genetic newborn screening for SMA on outcome.

**Methods:**

We provided clinical data from 43 SMA patients, identified via polymerase chain reaction of the *SMN1* gene from dried blood spots between January 2018 and January 2020 in Germany. Follow-up included neurophysiological examinations and standardized physiotherapeutic testing.

**Results:**

Detection of SMA with newborn screening was consistent with known incidence in Germany. Birth prevalence was 1:6910; 39.5% had 2 *SMN2* copies, 23% had 3 *SMN2* copies, 32.5% had 4 copies, and 4.5% had 5 copies of the *SMN2* gene. Treatment with SMA-specific medication could be started at the age of 14–39 days in 21 patients. Pre-symptomatically treated patients remained throughout asymptomatic within the observation period. 47% of patients with 2 *SMN2* copies showed early, presumably intrauterine onset of disease. These patients reached motor milestones with delay; none of them developed respiratory symptoms. Untreated children with 2 *SMN2* copies died. Untreated children with 3 *SMN2* copies developed proximal weakness in their first year. In patients with ≥ 4 *SMN2* copies, a follow-up strategy of “watchful waiting” was applied despite the fact that one of them was treated from the age of 6 months. Two infant siblings with 4 *SMN2* copies were identified with a missed diagnosis of SMA type 3.

**Conclusion:**

Identification of newborns with infantile SMA and prompt SMA-specific treatment substantially improves neurodevelopmental outcome, and we recommend implementation in the public newborn screening in countries where therapy is available. Electrophysiology is a relevant parameter to support the urgency of therapy. There has to be a short time interval between a positive screening result and referral to a therapy-ready specialized treatment center.

**Supplementary Information:**

The online version contains supplementary material available at 10.1186/s13023-021-01783-8.

## Introduction

Spinal muscular atrophy (SMA) is the most common neurodegenerative disease in childhood. Before pharmacological treatment became available, SMA was the most frequent monogenic cause of death in infancy. There are different types of severity, and the classification is based on the natural history of the disease. Children with SMA type 1 are unable to sit and most often die from respiratory failure in their first two years of life. Children with SMA type 2 show first symptoms between 6 and 18 months of age, achieve the ability to sit but not to walk, show moderate respiratory dysfunction and experience scoliosis. Children with SMA type 3 show onset of symptoms after ambulation has been acquired, however, often only transitorily. Children with SMA type 0 who show severe weakness and respiratory insufficiency at birth, or patients with SMA type 4 who show onset of disease late in adulthood account for less than 2% of cases.

A homozygous deletion in the *SMN1*gene, localized on chromosome 5q, encoding the “survival motor neuron” (SMN) protein, is responsible for the autosomal recessive disorder in more than 95% of cases [2]. Reduced levels of SMN protein result in motor neuron death in the spinal cord. In humans, there is a paralogous gene termed *SMN2* that differs from *SMN1* by only a few nucleotides. A critical c.840 C > T transition results in aberrant splicing, excluding Exon 7. Only 5–10% of functional protein result from transcription of *SMN2*. Thus, the severity of symptoms in SMA largely depends on the *SMN2* copy number, however, there are other genetic modifiers [3]. Patients with 2 copies of *SMN2* most often develop SMA type 1 and less frequently type 2; patients with 3 copies of the *SMN2* gene most commonly develop SMA type 2 but can also develop type 1 or type 3; and patients with 4 or more copies of the *SMN2* gene usually develop type 3 or type 4 SMA. Expression of SMN protein in spinal cord samples is highest during early stages of development [4].

The incidence rate in newborns is usually 1:6.000 to 1:11.000 [2]. Pooled data from neuromuscular centers, genetic institutes and patient registries revealed an incidence of 1:7352 in Germany in 2014 [5].

Available treatment for SMA includes *SMN2* splicing modifiers and gene replacement therapy, and both have shown to alter the course of SMA in humans [6–8]. Nusinersen (Spinraza®), an antisense oligonucleotide and intrathecal splicing modifier, was approved by the FDA in 2016 and by the EMA in 2017 for all subtypes of 5q-SMA. The adeno-associated virus vector-based gene therapy onasemnogene abeparvovec xioi (Zolgensma®) was approved by the FDA in July 2019 for 5q-SMA in children < 2 years and by the EMA in May 2020 for 5q-SMA in patients with 2 or 3 *SMN2* copies. The oral splicing modifier Evrysdi (Risdiplam®) was approved by the FDA in July 2020 and serves to treat patients two months of age and older with 5q-SMA. Several attempts at SMN-independent therapies are currently underway [3].

Given the pathophysiology of the disease and data from pre-clinical models demonstrating rapid death of motor neurons [9, 10], early intervention is mandatory for a better outcome [11]. Experts agree that newborn screening (NBS) should be established, and first pilot projects for a genetic NBS for SMA are underway [1, 12–16]. In the United States, NBS for SMA was added to the recommended uniform screening panel in 2018, and 33 states have been conducting NBS for SMA since November 2020. In Germany, SMA is going to be implemented in the general screening in 2021.

The objective of this study was to assess the impact of newborn screening for SMA on clinical and electrophysiological outcome.

## Methods

Screening for SMA was initially performed as part of a pilot project on genetic screening for cystinosis and SMA [1]. Since May 2019, screening for SMA has continued on the coauthors’ own initiative of this work. Quantitative PCR of DNA extracted from DBS was performed to screen for homozygous deletion of exon 7 [17]; heterozygous carriers were not detected. The screening laboratory covers approximately 78% of newborns in Bavaria and 37% in North Rhine-Westphalia. The number of initially non-participating hospitals declined from six to one during the projects. One clinic formally declared willingness to participate, but NBS cards were uniformly sent without order for SMA screening.

In case of a positive screening result, the respective treatment center for SMA (Munich, Essen or Münster) was informed by the screening laboratory [1]. The parents were contacted by the treatment center and an immediate appointment, usually on the following day, was offered for information and confirmation of diagnosis and *SMN2* copy number determination. The parents agreed to this procedure for reasons of data protection in the screening information.

Children born between January 2018 and January 2020 were included in this follow-up, with data collection ending in April 2020.

Data collection was performed as part of a prospective cohort study. Procedure after referral to the specialized neuromuscular centers was designed according to the standard of care for all SMA patients in our centers. Confirmation of the homozygous deletion of exon 7 of the *SMN1* gene and determination of the *SMN2* copy number by MLPA were performed using a new, whole blood sample in a collaborative laboratory for human genetics. The methodology was changed to a modernized version of the original MLPA kit in February 2019. After misanalysis was uncovered in one patient, all samples were re-analyzed with the newer kit in two independent laboratories.

The study protocol provided for a treatment decision in accordance with the recommendations of the “American SMA NBS Multidisciplinary Working Group,” published in 2018 [18]: Immediate treatment with Nusinersen was recommended to children with 2 and 3 *SMN2* copies, and a “watchful waiting” strategy to children with ≥ 4 copies. Every 2–4 months, patients underwent regular standardized neuropediatric examination, comprising electrophysiological exams, the “Children’s Hospital of Philadelphia Infant Test of Neuromuscular Disorders” (CHOP INTEND), a reliable measure for patients with SMA, comprising 16 items for evaluation of motor skills [19] and the “Hammersmith Infant Neurological Examination Sect. 2” (HINE-2), an assessment tool for evaluating motor milestones in infants with SMA, comprising eight sections [20].

Children with normal muscle tone, a CHOP INTEND score of > 35 points, an ulnar CMAP amplitude > 1 mV (this refers to the first examination, age range 6–14 days), and no deterioration in their first 4 weeks of life were considered pre-symptomatic.

The local ethics committee of the participating universities (project no. 18–269) approved the study.

## Results

### Demographics and baseline measures

87% of DBS cards were marked to opt for SMA screening, corresponding to a total number of 297.163 investigated samples from the two projects mentioned above. There were 43 cases detected with a homozygous deletion of the *SMN1* gene, resulting in a birth prevalence of 1:6910. Positive results were reported to the neuromuscular center on median day 6 of life (range 3–9 days, all within normal procedure). A second blood sample was collected on median day 8 of life (range 6–14 days, all within normal procedure). All positive tests in the NBS were confirmed by MLPA. Confirmation of diagnosis and determination of the *SMN2* copy number were available at median day 14 of life (range 9–23 days, delay in one child due to loss of sample in public mail). *SMN2* copy number determination revealed three false results, discovered by repeated analysis with an improved kit: Twice the analysis incorrectly revealed 4 instead of 5 *SMN2* copies and once the analysis incorrectly revealed 4 instead of 3 *SMN2* copies. Finally, seventeen patients (39.5%) had 2 *SMN2* copies, ten patients (23%) had 3 *SMN2* copies, fourteen patients (32.5%) had 4 *SMN2* copies, and two patients (4.5%) had 5 copies of the *SMN2* gene. Until now, no SMA case missed by NBS has been detected.

No child showed any signs of respiratory involvement or bulbar weakness immediately after birth. Nine patients with 2 *SMN2* copies and all patients with 3 or more *SMN2* copies were asymptomatic in the first examination and had ulnar CMAPs > 1 mV. Eight patients with 2 *SMN2* copies had early signs of an already active disease process. Ulnar CMAPs were < 1 mV in five children, three of them additionally had a CHOP INTEND Score of ≤ 35 at first examination and one of them developed severe muscular weakness of the lower extremities at the age of 2 weeks. One more patient, without initial electrophysiologic examination, declined severely at the age of 2 weeks. One child had isolated low CMAP amplitudes but showed no clinical deterioration under immediate treatment, though. Two children showed a decline in muscle strength in the legs during the first weeks of life despite ulnar CMAPs of 1.2 and 1.1 mV, respectively. The median interval between confirmation of diagnosis and initiation of treatment was 6.5 days (range 1–16 days, delay in one child due to the need for health insurance coverage). For details see Additional file 1: Table S1.

### Outcome in treated children with 2 SMN2 copies

**Fig. 1:**
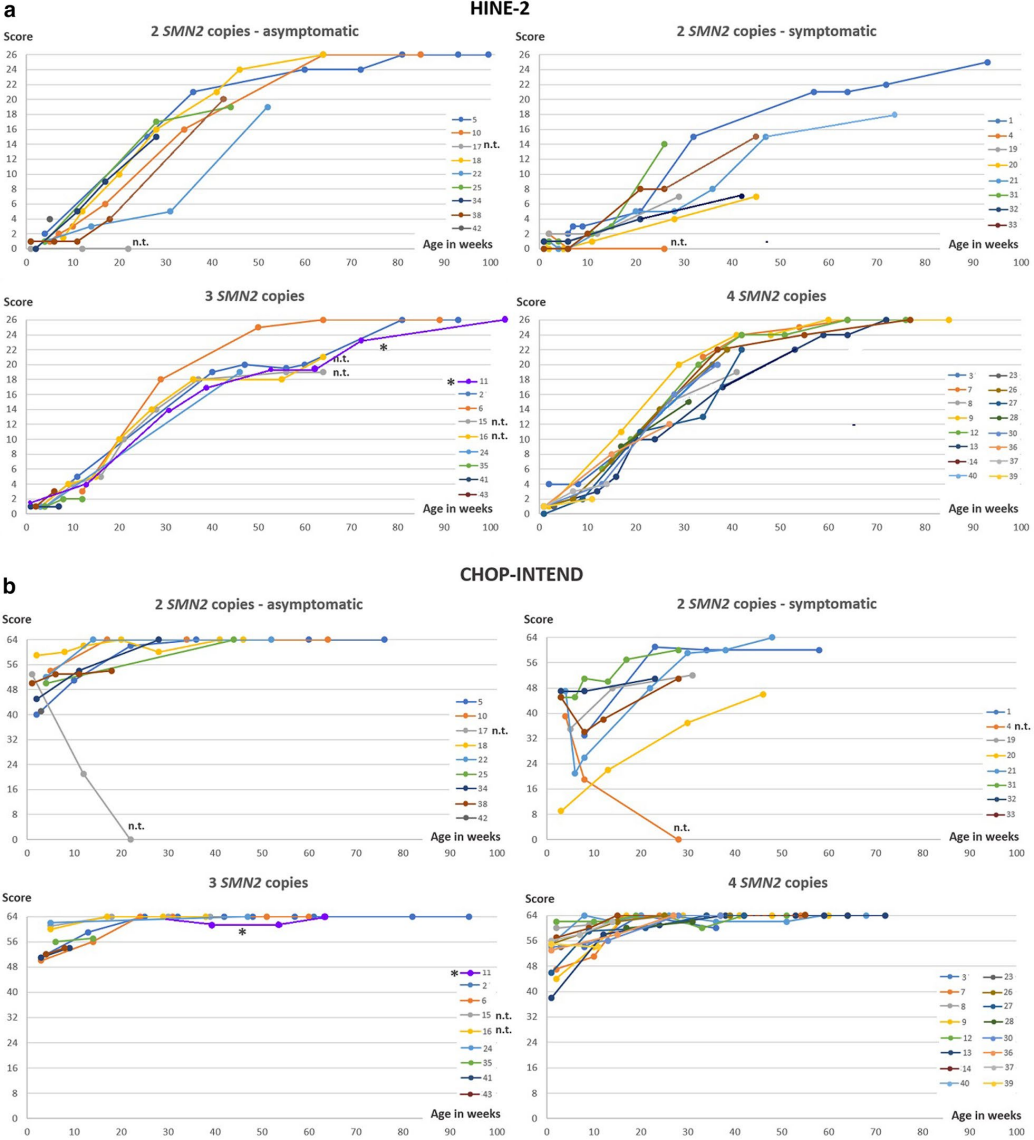

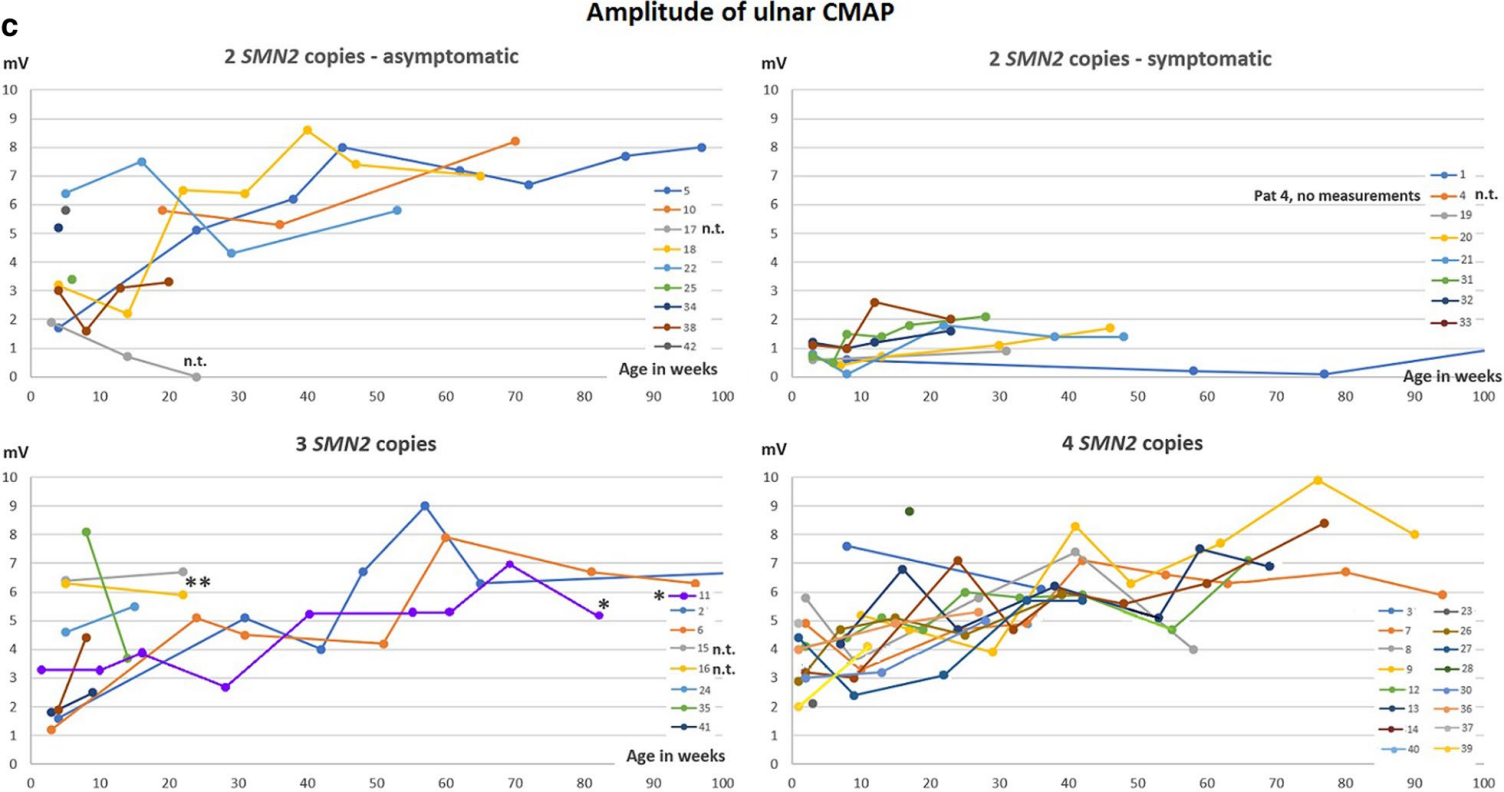
Course of **a** HINE-2, **b** CHOP INTEND and **c** Ulnar CMAPs with increasing age. n.t. = not treated. * Pat 11, symptomatic age 8 months, treated from the age of 10 months. **Pat 15 and 16, not treated: Further measurements were refused by the parents

**Fig. 2:**
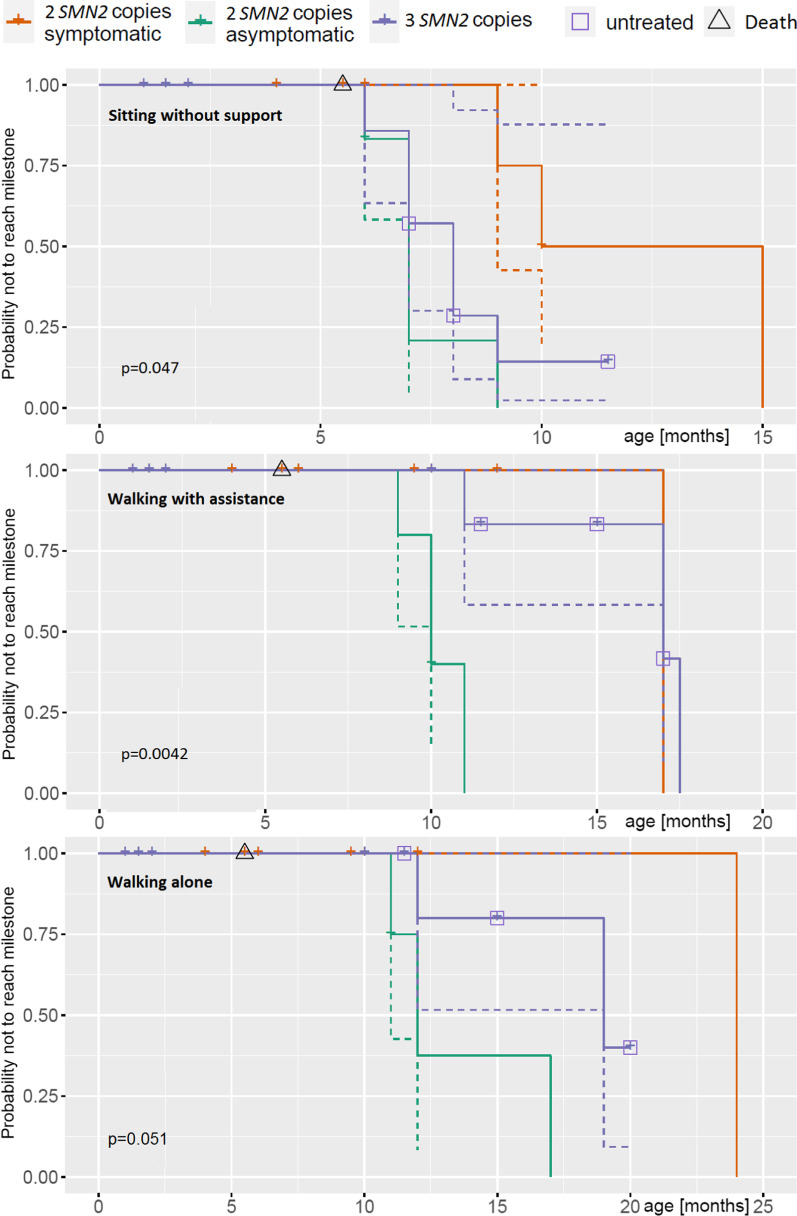
x-axis: age in months (either at the time of the time reaching the respective milestone or at age of last presentation). y-axis: probability of NOT reaching the milestone. log-rank test: test for equality of the three different curves. The P-value (Log-Rank-Test) < 0.05 indicates that the three curves differ significantly from each other. Solid lines: Result curves. Dashed lines in corresponding colors: confidence interval

Fifteen of seventeen children with 2 *SMN2* copies were treated with Nusinersen from age 14–39 days. Eight children, who were considered to be pre-symptomatic, have remained symptom-free so far and achieved normal motor milestones. Seven children already had overt or subtle signs of disease in their first days or weeks of life. In all of them, CHOP INTEND and HINE-2 results improved under therapy (Fig. 1a). However, motor milestones were delayed in comparison to initially asymptomatic children (Fig. 2, Additional file 1: Table S1). No respiratory involvement has occurred in any early treated patient with 2 *SMN2* copies and no child has developed orthopedic complications like scoliosis or contractures, or feeding by gastral tube so far.

### Outcome in untreated patients with 2 SMN2 copies

Two patients with 2 *SMN2* copies could not be treated. One family decided against the treatment offered considering the severity of the natural history and the limited data on treatment success in early-detected SMA. The other patient was the child of a Romanian family without permanent German residence or citizenship, so therapy could not be offered due to lack of reimbursement of the cost-intensive medication. The latter had already experienced symptoms in the first weeks of life, the former remained clinically asymptomatic until the age of 3 months, during which time a rapid deterioration started. Both children died at the age of 5.5 months due to respiratory failure.

### Outcome in treated children with 3 SMN2 copies

Six of ten children with 3 *SMN2* copies were treated with Nusinersen from age 20–29 days. Median follow-up period regarding motor milestones was 13 months (average 13.2 months, range 5–24 months) (Fig. 2). All treated children with 3 *SMN2* copies remained asymptomatic, as far as the observation period allows to state (since some 3 *SMN2* copy infants have not become symptomatic until 12–15 months of age). One patient with joint laxity but without neurophysiological findings of SMA showed minimal delay of motor milestones. All other treated patients in this cohort remained asymptomatic with normal milestone achievements and no respiratory manifestation during the observation period.

### Outcome in untreated patients with 3 SMN2 copies

Four patients with 3 *SMN2* copies could not be treated immediately; for twins, parents refused treatment. The discussion with the treating physicians revealed that, on the one hand, religion was one reason, on the other hand it was due to the fear of the invasiveness of treatment with Nusinersen which was the only approved SMA-specific drug at that time. Proximal weakness occurred in both children at the age of 11 months. One family initially refused any medical contact when they were informed about the positive screening result. Motor deterioration was noticed at the age of 6 months and the child was then seen in the treatment center for the very first time. Both families did stick with their decision against pharmacological treatment when symptoms occurred. One child was misdiagnosed with 4 *SMN2* copies and developed proximal weakness at the age of 8 months.

### Therapy and outcome in patients with ≥ 4 SMN2 copies and secondary diagnosis for siblings

Fourteen patients had 4 *SMN2* copies and two patients had 5 *SMN2* copies. Median follow-up period regarding motor milestones (Fig. 2) was 13.2 months (average 13.0 months, range 1.5–26 months). In one child with 4 *SMN2* copies, the parents opted for treatment due to a positive family history, and Nusinersen was applied from the age of six months. All patients did not show any symptoms until their last examination.

For details on outcomes, ethnic background, and family history, see Additional file 2: Table S2. Two families with newborns with 4 *SMN2* copies reported during follow-up that the respective 5-year-old and 6-year-old brother had unclear motor symptoms. While one brother had an unsteady gait and a tremor with onset at the age of 3 years, the other one tended to walk on tiptoe and showed muscular fatigue from the age of 3 years. The former had been diagnosed as congenital ataxia and the latter as clumsiness. A homozygous deletion in the *SMN1* gene proved the diagnosis of SMA 3 and treatment was initiated in both. In the two screened index patients, start of treatment within the first year of life irrespective of the clinical status is under discussion with the parents. Three patients with 4 *SMN2* copies were lost of follow-up at different time points (Fig. 2).

### Electrophysiology

Ulnar CMAP amplitudes of all patient groups are shown in Fig. 1c; children with CMAP < 1 mV were considered directly symptomatic. However, all other children found to be early symptomatic by clinical parameters also had ulnar CMAP amplitudes < 1.5 mV, whereas all children who were pre-symptomatic by clinical criteria showed amplitudes > 1.5 mV. After treatment, CMAPs increased in the early symptomatic group but did not reach the level of the asymptomatic children.

## Discussion:

This study shows that the identification of newborns with infantile SMA will lead to a substantial improvement in neurological outcome if prompt SMA-specific medication can be provided. The fact that all pre-symptomatically treated patients, even with 2 *SMN2* copies, have shown normal motor development so far, is a clear argument that pre-symptomatic therapy may prevent the death of motor neurons. The opposite was also true, all untreated children in this study with 2 or 3 *SMN2* copies have consistently developed infantile SMA.

After 2 years of clinical follow-up, we found that a substantial proportion of patients with 2 *SMN2* copies had an active disease process within days of birth. Nearly half of them showed signs of motor neuron dysfunction in their first weeks of life. Of particular note are the subtle reduction of muscular strength or low ulnar CMAPs, which would have been missed at the time during a routine examination. NBS avoided a diagnostic delay [21, 22] and our clinical results suggest that motor development in this group of patients is possible, even if the diagnosis is made at the time of an already incipient motor neuron process.

Our findings are consistent with Biogen’s phase 2 study NCT02386553 (NURTURE) which enrolled 25 patients with early-diagnosed SMA and 2 or 3 copies of the *SMN2* gene shortly after birth [11]. Their preliminary data shows that the effect will last at least up to 4 years [11]. Avexis’s phase 3 study NCT03505099 (SPR1NT), which has evaluated the safety and efficacy of intravenous onasemnogene abeparvovec-xioi in a corresponding patient cohort, offers a follow-up period that is not quite as long, but shows similarly promising results. In both studies, no treated child has developed respiratory involvement so far or died. In Biogen’s phase 3 study NCT02193074 (ENDEAR), which tested the clinical efficacy of Nusinersen versus Sham Control in infantile-onset SMA in symptomatic children, ventilator-free survival was 61% only.

Our data suggests that the diagnostic value of CMAPs at first investigation is highly relevant. All children who eventually turned out to be early symptomatic had low amplitudes. The most important predictive value of the CMAP amplitude seems to be in capturing those children who are asymptomatic at a clinical level but symptomatic at an electrophysiological level especially with 2 copies of the *SMN2* gene. Low CMAPs in a clinically unremarkable patient must be considered an alarm sign, and delay of therapy should not be accepted under any circumstances. A cut-off of 1 mV, which was adopted from the inclusion criteria for the NURTURE study, seems too low to exclude early disease onset with certainty. Based on our ulnar CMAP amplitude graphs, 1.5 mV could be a more reliable cut-off. It seems also possible that there could be subgroups of treatment responders identified; however, the current observation period of the symptomatic group is too short to make any final statements in this regard; a re-analysis after one or two years will be useful.

The handling of patients with ≥ 4 copies of the *SMN2* gene is still a matter of debate [23]. There is an ongoing discussion whether and when patients with 4 *SMN2* copies will become symptomatic, as well on the burden of early treatment in a potential late-onset disease versus the risk of delayed diagnosis with irreversible motor damage. This dilemma led to a missing consensus of the SMA NBS Multidisciplinary Working Group in 2018 and 2020. Finally, the group modified the recommendations and voted for a treatment of children with 4 *SMN2* copies and a strategy of watchful waiting for those with 5 *SMN2* copies [18, 24], but still of different opinions of the individual experts. Our data are not yet able to answer this question conclusively. Long-term data will be needed and the cohort will be monitored closely accordingly. However, the fact that in the group of patients with 4 *SMN2* copies three close relatives of the children with the same genotype had developed SMA type 3 in early childhood is an indication that 4 *SMN2* copies do not necessarily predict late-onset SMA and that treatment in childhood may prevent the manifestation of SMA type 3.

The estimation of the *SMN2* copy number poses some methodological problems. In up to 45% of cases, retesting is known to lead to a miscall of the initially determined copy number [25], a problem mainly in patients ≥ 4 copies, depending on the quality of the DNA, but although relevant in all other patients. In this study-cohort, one child with 3 *SMN2* copies was initially diagnosed with 4 *SMN2* copies and became symptomatic. Two children were diagnosed with 4 *SMN2* copies initially and then turned out to have 5 copies. This highlights the necessity of confirming the copy number in a second laboratory, as the treatment algorithm is based on the *SMN2* copy number.

The basic criteria for a newborn screening [26] are given; sensitivity and specificity are at a high level, and most parents opted for a screening. A former study showed that there is a wide acceptance of SMA NBS in the British population [27]. Adding a genetic screening to NBS had no negative effect on the overall acceptance of NBS. A critical issue is to convince obstetricians that the benefits of SMA NBS outweigh the additional workload associated with providing additional informed consent for genetic screening.

In terms of cost factors for SMA-NBS, the implementation of the screening into the existing NBS structures is a decisive factor in addition to the laboratory costs for the test. PCR itself is comparatively inexpensive, but screening means a very high number of tests. On the other hand, avoiding higher morbidity is a relevant cost-reducing factor for the health care system. So far, none of the children in this study has incurred additional costs other than medication. Studies on medical costs put the cost per patient in Germany at €70.566 in 2013 [28]. Drug-independent median health care costs for treated children with SMA type 1 in the U.S. were put at $92,618 [29]. Thus, NBS substantially reduces subsequent health care costs. Better quality of life can be expected due to the less severe course of the disease. It is known that high social and economic costs are also due to the relevant psychosocial burden of patients with symptomatic SMA and their caregivers [30].

We strongly recommend that newborn screening for SMA ought to become universal in countries that provide SMA-specific medication. When implementing SMA in a public screening program, it must be ensured that affected children are treated, and treated on time without much delay due access problems. It must be assumed that asymptomatic neuronal injury is likely to occur in utero [4] and that additional 2–6 weeks to start treatment will worsen prognosis. Of particular importance is the referral to a specialized center without loss of time, which is why we recommend direct contact between screening laboratory and SMA center to inform the parents in time [1]. This saves time, is likely to reduce the stress of the affected families and avoids the unnecessary consultation of medical centers that are either not specialized or unable to provide pharmacotherapy.

Even in countries with a less robust health care system, a fast-track procedure with confirmation of diagnosis within less than one week and initiation of prompt treatment should be implemented in case of positive SMA-NBS findings. The benefit of NBS for the relevant number of early symptomatic children will decrease decisively if time gets lost. SMA is present worldwide. The incidence rate is usually given as 1:6000–1:11.000 [2] and was last reported to be in Taiwan 1:17.000 [16]; it is basically a “rare disease”. The method for SMA screening is highly specific. Accordingly, the necessity of these structures applies for a manageable amount of patients.

## Limitations

This is a descriptive study and not a randomized case–control study. In view of the clarity of the improvements in outcome, e. g. in comparison to the ENDEAR Study [7], such a study does no longer seem ethically justifiable, at least in countries that have pharmacotherapy available for all patients. However, questions remain, especially regarding the right timing of treatment for patients with 4 *SMN2* copies.

The sample size of this study limits a more in-depth statistical analysis. The follow-up duration is comparatively short, and data collection is not entirely uniform. These factors are related to the novelty of the subject, but long-term data will be needed in the future.

All patients in this study were treated with Nusinersen, as gene therapy had not yet been approved by the EMA at the time treatment started and the observation period of the included patients. As gene therapy is now available in Germany, it may represent the first treatment option for a part of patients detected by NBS, and it is possible that the different pharmacokinetics could have an impact on outcome, particularly in very early symptomatic patients.

The introduction of screening without subsequent treatment options presents a significant ethical dilemma and is fundamentally at odds with the general screening guidelines of the WHO. Even in this study, conducted in a country with usually universal health insurance coverage, the diagnosis of SMA was made in a child who was not eligible for treatment.

Regarding the question of how to proceed in countries where there is no health insurance coverage for all citizens, this study can give no other answer than that such insurance coverage seems essential at least for the total group of SMA patients.

## Summary and conclusion

Prompt treatment after genetic NBS for SMA substantially improves outcome in infantile SMA. Sensitivity [1] and specificity are high and there have been no false-positive or false-negative results so far. Data suggests that patients in whom the disease does not become clinically manifest are not identified, as comparison with existing German data confirmed that NBS did not lead to a relevant increase in incidence. The cost–benefit assessment for the expenses to the general public appears favorable. The optimal time to start treatment for patients with 4 *SMN2* copies cannot be determined by these data. If a watchful waiting strategy is favored, double determination of the *SMN2* copy number should be considered, as the method still does not appear to be completely reliable.

We strongly recommend the implementation of a genetic SMA screening in existing NBS structures where SMA-specific therapy is available. The time interval between a positive screening sample and referral to a therapy-ready specialized treatment center has to be short. Electrophysiology is a relevant parameter to support the urgency of therapy in children with presumably already intrauterine-onset of motoneuron damage. Identifying SMA patients without guaranteeing therapy presents an ethical dilemma.

## Supplementary Information

**Additional file 1**. **Table S1**: Age at first consultation at the treatment center or at collection of thesecond blood sample, discrepancy in SMN2 estimation, early-onset symptoms, and initial resultsfrom electrophysiology and CHOP INTEND. Y = years, mo = months, d = days, neg = negative,pos = positive.

**Additional file 2**. **Table S2**: Patient’s family history, ethnical background, and outcome details. = years, mo = months, d = days, neg = negative, pos = positive.

## Data Availability

All data from CHOP INTEND, HINE-2 and electrophysiology studies can be shared and is available at the author’s institution in Munich, Dr. v. Haunersches Kinderspital, Lindwurmstr. 4, 80337 München, Germany.

## Abbreviations

SMA: Spinal muscular atrophy
NBS: Newborn screening
DBS: Dried blood spots
CHOP INTEND: The Children’s Hospital of Philadelphia Infant Test of Neuromuscular Disorders
HINE-2: Hammersmith Infant Neurological Examination – Sect. 2
PCR: Polymerase chain reaction
MLPA: Multiplex ligation-dependent probe amplification
SMN: Survival motor neuron
CMAP: Compound muscle action potential
