# Supplementary material for: Newborn screening for spinal muscular atrophy in Germany: clinical results after 2 years
Source: Orphanet J Rare Dis. 2021 Mar 31;16:153. doi: 10.1186/s13023-021-01783-8 (PMC8011100; doi:10.1186/s13023-021-01783-8)
Supplement: Supplementary file 1 — Additional file 1. Table S1: Age at first consultation at the treatment center or at collection of thesecond blood sample, discrepancy in SMN2 estimation, early-onset symptoms, and initial resultsfrom electrophysiology and CHOP INTEND. Y = years, mo = months, d = days, neg = negative,pos = positive. [file 13023_2021_1783_MOESM1_ESM.docx]

| **Patient Nr.** | ***SMN2* copy (n)** | **Age at first consultation in treatment center/collection of second blood sample (age in days)** | **Discrepancy in SMN2 estimation (n)** | **Clinical symptoms during first 4 weeks of life** | **First CHOP INTEND (age in days)** | **First ulnar CMAP  (age in days)** |
| --- | --- | --- | --- | --- | --- | --- |
| 1 | 2 | 11 | - | Hypotonia of legs age 4 weeks | 33 (39) | 0.6 (39) |
| 2 | 3 | 9 | - | no | 52 (15) | 1.6 (15) |
| 3 | 4 | 13 | - | no | 54 (53) | 7.6 (53) |
| 4 | 2 | 14 | - | General weakness and respiratory dysfunction age 2 weeks | 39 (14) | n.a. |
| 5 | 2 | 8 | - | no | 40 (13) | 1.7 (13) |
| 6 | 3 | 10 | - | no | 50 (10) | 1.2 (10) |
| 7 | 4 | 10 | - | no | 47 (16) | 4.9 (16) |
| 8 | 4 | 6 | - | no | 60 (12) | 5.8 (12) |
| 9 | 5 | 9 | 4 >> 5 | no | 44 (12) | 5.2 (12) |
| 10 | 2 | 10 | - | no | 54 (53) | n.a. |
| 11 | 3 | 10 | 4 >> 3 | no | 55 (10) | 3.4 (10) |
| 12 | 5 | 8 | 4 >> 5 | no | 62 (13) | 4.1 (13) |
| 13 | 4 | 8 | - | no | 38 (8) | 4.2 (8) |
| 14 | 4 | 9 | - | no | 57 (15) | 3.2 |
| 15 (Twin) | 3 | 17 | - | no | 61 (22) | 6.4 |
| 16 (Twin) | 3 | 8 | - | no | 60 (22) | 6.3 |
| 17 | 2 | 7 | - | no | 53 (7) | 1.9 (7) |
| 18 | 2 | 7 | - | no | 59 (6) | 3.2 (6) |
| 19 | 2 | 8 | - | Hypotonia of legs age 2 weeks | 35 (8) | 0.6 (8) |
| 20 | 2 | 9 | - | General weakness with birth | 9 (9) | 0.4 (9) |
| 21 | 2 | 8 | - | Severe weakness of legs age 2 weeks | 48 (8) | 0.8 (8) |
| 22 | 2 | xx | - | no | 58 (8) | 6.4 (8) |
| 23 | 4 | 9 | - | no | 54 (24) | 2.1 (24) |
| 24 | 3 | xx | - | no | 62 (20) | 4.6 (20) |
| 25 | 2 | xx | - | no | 50 (27) | 3.4 (27) |
| 26 | 4 | 8 | - | no | 55 (8) | 2.9 (8) |
| 27 | 4 | 9 | - | no | 46 (9) | 4.4 (9) |
| 28 | 4 | 9 | - | no | 60 (121) | 8.8 (121) |
| 29 | 3 | Appointment for consultation and confirmation diagnostics was refused | - | ? | - | - |
| 30 | 4 | 8 | - | no | 54 (12) | 3 (12) |
| 31 | 2 | 8 | - | no | 45 (6) | 0.7 (6) |
| 32 | 2 | 7 | - | Hypotonia of legs age 3 weeks | 47 (7) | 1.2 (7) |
| 33 | 2 | 8 | - | Hypotonia of legs age 4 weeks | 45 (8) | 1.1 (8) |
| 34 | 2 | 11 | - | no | 45 (15) | 5.2 (15) |
| 35 | 3 | 8 | - | no | 56 (28) | 8.1 (28) |
| 36 | 4 | 7 | - | no | 53 (7) | 4.0 (7) |
| 37 | 4 | 9 | - | no | 56 (9) | 4.9 (9) |
| 38 | 2 | 8 | - | no | 50 (11) | 3.0 (11) |
| 39 | 4 | 9 | - | no | 55 (9) | 2.0 (9) |
| 40 | 4 | 9 | - | no | 56 (82) | 1,6 (82) |
| 41 | 3 | 6 | - | no | 51 (10) | 1.8 (10) |
| 42 | 2 | 9 | - | no | 41 (19) | 5.8 (19) |
| 43 | 3 | 6 | - | no | 52 (11) | 1.9 (11) |
