# Supplementary material for: Newborn screening for spinal muscular atrophy in Germany: clinical results after 2 years
Source: Orphanet J Rare Dis. 2021 Mar 31;16:153. doi: 10.1186/s13023-021-01783-8 (PMC8011100; doi:10.1186/s13023-021-01783-8)
Supplement: Supplementary file 2 — Additional file 2. Table S2: Patient’s family history, ethnical background, and outcome details. = years, mo = months, d = days, neg = negative, pos = positive. [file 13023_2021_1783_MOESM2_ESM.docx]

| **Patient Nr.** | ***SMN2* copy (n)** | **Family history** | **Country of parent’s Origin** | **Consang-inity of parents** | **Age at first dose of medication** | **Best motor milestone so far achieved** | **Detoriation** | **Deceased** | **Age at last visit** |
| --- | --- | --- | --- | --- | --- | --- | --- | --- | --- |
| 1 | 2 | neg | Germany | no | 39 d | Walking alone 24 mo | - | - | 26 mo |
| 2 | 3 | neg | Germany | no | 24 d | Walking alone 19 mo | - | - | 23 mo |
| 3 | 4 | neg | Romania | no | - | Sitting without support 8 mo | - | - | 8 mo |
| 4 | 2 | neg | Romania | yes | Lack of reim-bursement | - |  | 5.5 mo | - |
| 5 | 2 | neg | Germany | no | 15 d | Walking alone 17 mo running 22 mo | - | - | 26 mo |
| 6 | 3 | neg | Germany | no | 24 d | Walking alone 12 mo, running 20 months | - | - | 21 mo |
| 7 | 4 | neg | Germany | no | - | Walking alone 12 mo | - | - | 22 mo |
| 8 | 4 | neg | Albania | no | - | Walking alone 13 mo | - | - | 13 mo |
| 9 | 5 | neg | Germany | no | - | Walking alone 14 mo | - | - | 20 mo |
| 10 | 2 | neg | Turkey | yes | 35 d | Walking alone 11 mo | - | - | 20 mo |
| 11 | 3 | neg | Germany/Austria | no | 10 mo | Walking alone 25 mo | 8 mo proximal weakness |  | 26 mo |
| 12 | 5 | neg | Germany | no | - | Walking alone 13 mo |  |  | 18 mo |
| 13 | 4 | neg >> positive (misdiagnosed brother) | Germany | no | - | Walking with assistance 13 mo | - | - | 17 mo |
| 14 | 4 | neg | Germany | no | - | Walking alone 14 mo, running 17 mo | - | - | 17 mo |
| 15 (Twin) | 3 | neg | Bulgaria | yes | Refused by parents | Sitting without support 8 mo | 11 mo proximal weakness | - | 15 mo |
| 16 (Twin) | 3 | neg | Bulgaria | yes | Refused by parents | Sitting without support 8 mo | 11 mo proximal weakness | - | 15 mo |
| 17 | 2 | neg | Germany | no | Refused by parents | - | general weakness and respiratory dysfunction age 12 weeks | 5.5 mo | 3 mo |
| 18 | 2 | neg | Togo | no | 25 d | Walking alone 12 mo, running 20 months | - | - | 23 mo |
| 19 | 2 | pos | Syria | yes | 22 d | Sitting without support if seated with 9 months | - | - | 9,5 mo |
| 20 | 2 | neg | Germany | no | 17 d | rolling to both sides and sitting with support | - | - | 10 mo |
| 21 | 2 | neg | Poland | no | 15 d | Sitting without support 14 mo |  | - | 17 mo |
| 22 | 2 | neg | Azerbaijan | no | 17 d | walking with assistance 10 mo | - | - | 11 mo |
| 23 | 4 | neg | Germany | no | - | n.a. | - | - | n.a. |
| 24 | 3 | neg | Turkey | yes | 29 d | stands with support 10 mo | - | - | 10 mo |
| 25 | 2 | neg | Germany | no | 27 d | Sitting without support 9 mo | - | - | 10 mo |
| 26 | 4 | neg | Poland | no | - | standing with support 9 mo | - | - | 9 mo |
| 27 | 4 | neg | Germany | no | - | Walking alone 13.5 mo | - | - | 13.5 mo |
| 28 | 4 | pos | Turkey | yes | - | Sitting without support 7 mo | - | - | 7 mo |
| 29 | 3 | neg | Turkey | yes | Refused by parents | ------- | 6 mo proximal weakness | - | 11 mo |
| 30 | 4 | pos | Afghanistan | no | 6 mo | Sitting without support 7 mo | - | - | 7 mo |
| 31 | 2 | neg | Germany | no | 15 d | Sitting without support 11 mo | - | - | 12 mo |
| 32 | 2 | neg | Turkey | no | 14 d | Not yet sitting |  | - | 11 mo |
| 33 | 2 | neg | Germany | no | 16 d | Sitting without support 9 mo |  | - | 11 mo |
| 34 | 2 | neg | Russia | no | 15 d | sitting with support 6 mo | - | - | 6 mo |
| 35 | 3 | neg | UAE | yes | 28 d | Not yet sitting | - | - | 2 mo |
| 36 | 4 | neg >> positive (misdia-gnosed brother) | Germany | no | 6 mo | Not yet sitting | - | - | 6 mo |
| 37 | 4 | neg | Germany | no | - | Not yet sitting | - | - | 7 weeks |
| 38 | 2 | neg | Bangladesh | no | 15 d | Sitting without support 7 mo | - | - | 4 mo |
| 39 | 4 | neg | Germany | no | - | Not yet sitting | - | - | 11 weeks |
| 40 | 4 | neg |  | yes | - | Not yet sitting | - | - | n.a. |
| 41 | 3 | neg | Germany | no | 20 d | Sitting without support 7 mo | - | - | 8 mo |
| 42 | 2 | neg | Germany | no | 19 d | Not yet sitting | - | - | 7 weeks |
| 43 | 3 | neg | Germany | no | 20 d | Not yet sitting |  |  | 6 weeks |
